# Diabetogenic Potential of Ancestral and Modern Wheat Landraces

**DOI:** 10.3390/nu9080816

**Published:** 2017-07-28

**Authors:** Sandra V. Aguayo-Patrón, María E. Mejía-León, Ana M. Calderón de la Barca

**Affiliations:** Departamento de Nutrición y Metabolismo, Centro de Investigación en Alimentación y Desarrollo, A.C., Carretera a La Victoria, Km. 0.6, Hermosillo, Sonora 83304, Mexico; saguayop@gmail.com (S.V.A.-P.); esther.mejia83@gmail.com (M.E.M.-L.)

**Keywords:** ancestral and modern wheat, type 1 diabetes, gluten, *Triticum aestivum*, *Triticum turgidum*

Dear Editor,

We read with interest the article by Gorelick et al. [1], who assayed the diabetogenic potential of two ancestral wheat landraces (*Triticum turgidum* ssp. *dicoccoides* and spp. *dicoccum*), compared to a modern wheat cultivar (*T. aestivum*) in NOD mice. Although the general idea was great, we found some points that could confound the results.

According to Gorelick et al. [1], their “diabetogenic diet” composition was based on the one described by Funda et al. [2] (8.4% meat hydrolysate, 6.5% soybean protein, 1% milk protein, 3.2% corn starch, 2.5% wheat, and 1% oat proteins), just replacing wheat protein with an alternative protein source to make the “low diabetogenic diet”. However, the ingredients included in experimental diets of Gorelick et al. [1] were quite different from those of Funda et al. [2]. The latter research did not use cereal protein or maize starch, and they completed the total protein with meat protein, not with maize protein, as Gorelick et al. did.

The maize prolamins peptides are able to increase gut permeability and induce a less intense, but similar, immune response to that generated by gliadin peptides in biopsies of celiac patients or Caco-2 cells [3,4]. In addition, altered gut permeability and inflammation are risk factors for type 1 diabetes (T1D) [5], and T1D patients are prone to developing celiac disease [6]. Maize protein and maize meal in the “low diabetogenic diet” probably were confounders in the assay by Gorelick et al. [1]. They discuss that this could be a reason that explains the diabetogenic effect of non-wheat diet, but maize and maize meal were included in all five of the different tested diets, and there was no similar effect for the ancestral and modern wheat diets. Thus, the unexpected results in the non-wheat diet may be due to another source of variation.

Moreover, Gorelick et al. [1] included two *T. aestivum-*containing diets in the experiment, one cultured by them and another acquired in a commercial mill. The results indicated that the second one was diabetogenic, while the first one had no effect on NOD mice, similar to ancestral wheat diets. They named the diabetogenic diet the “standard diabetogenic wheat bread containing diet”, but it is unclear whether they included complete wheat grains, wheat protein or wheat bread made from the grains obtained in the commercial mill. Modern bread-making processes employ food additives as emulsifiers, which can increase gut permeability, affect gut microbiota, induce an inflammatory response and could increase the risk of T1D [7,8]. Thus, if wheat bread was used in the experiment, this could partially explain the diabetogenic effect of the mill-wheat diet compared to the lack of effect of the self-cultured wheat.

Another point is the use of whole wheat or isolated wheat protein in the experimental diets of Gorelick et al. [1]; although they describe in the Methods that wheat protein was used, in Table 1 [1], just “wheat” is written. The interpretation of the results could change if whole wheat was used; there are other components in addition to proteins that could interfere, avoiding the real identification of the diabetogenic potential of the tested diets. For example, dietary fiber is related to high production of short chain fatty acids by gut microbiota and, in turn, with the induction of immune tolerance [9].

The diagnosis of diabetes in NOD mice was supported by glucose in urine and fasting blood glucose evaluations, with 150 mg/dL and 130 mg/dL as cutoff values, respectively. Following this, Gorelick et al. [1] declared a null incidence of T1D in the groups that received the diets containing the ancestral wheat landraces and the modern wheat landrace cultured by themselves (groups 3 to 5). However, fasting blood glucose results at the end of the experiment in groups 3 and 5 (Figure 3) [1] had standard errors that reached or surpassed 130 mg/dL, indicating that any subject in these groups could accomplish the criteria for T1D diagnosis.

Finally, there is no information about the quantity of animals analyzed for IL-10 and IFN-γ; were all 50 animals analyzed, or it was a subsample? Currently intra-group variability for these markers is large in different animal models and human beings, quite different from the ones in Figures 5 and 6 [1].

Perhaps experimental details such as the real composition of the diets, describing each component or ingredient and their proportions, could help to understand these quite interesting results, and to use them as base for future experiments or even for treatments to prevent autoimmune diseases.
